# Palliative Treatment of Asthma

**Published:** 1861

**Authors:** T. L. Pridham

**Affiliations:** Bideford, North Devon


### ON THE PALLIATIVE TREATMENT OF ASTHMA.

By T. L. PRIDHAM, Esq., Bideford, North Devon.

The first on the list is stramonium, the fumes of which may be collected in an inverted glass bowl with a narrow mouth; the bowl being charged to its full is placed under the mouth of the patient, who is directed to inhale to the fullest extent in his power the smoke which has been collected in the bowl, taking care to hold his head away from the bowl, when an expiration takes place. Chloroform, both taken internally or inhaled, is a powerful remedy, but must be employed with caution, and never administered except by a medical attendant. The fumes of nitre paper in a state of ignition, well inhaled, is often a valuable remedy. Care should be taken to procure the best prepared from a good chemist. Chloric ether and the tincture of the lobelia inflata will occasionally relieve.—Bicarbonate of soda, as well as chlorate of potass, given in full doses, I have frequently seen produce a good effect. Again, I have seen repeated doses of sulphurate of alum procure relief, the powder being allowed to dissolve on the tongue before it is swallowed in ten grain doses. I have also seen the fumes of tobacco, inhaled as I have recommended in the use of stramonium, relieve, when other remedies have failed; but I do not like this remedy, it produces such deadly faintness and nausea. Small drinks of the best Mocha coffee, made strong, will often procure relief. On two occasions, when every other remedy failed, I succeeded in procuring almost instant relief, by injecting two grains of morphine and a drachm of tincture of assafœtida. These were cases where mental distress appeared to be the exciting cause.

I have often sat at the bed-side of one, suffering from the severest form of the disease, watching with great anxiety the result of prescribed remedies, and it has not unfrequently happened that many have been tried without relief, the patient all this time gasping for life with sufferings the most intense, when relief has at length come from a remedy apparently the most unlikely to procure it—so capricious is the disease, and so uncertain the remedy in asthma cases of this particular character.—*Brit. Med. Jour. Dec.* 29, 1860, *p.* 1000.

